# Undermining during cutaneous wound closure for wounds less than 3 cm in diameter: a randomized split wound comparative effectiveness trial

**DOI:** 10.1007/s00403-021-02280-5

**Published:** 2021-09-21

**Authors:** Jayne Joo, Aunna Pourang, Catherine N. Tchanque-Fossuo, April W. Armstrong, Danielle M. Tartar, Thomas H. King, Raja K. Sivamani, Daniel B. Eisen

**Affiliations:** 1 Department of Dermatology, University of California, Davis, School of Medicine, 3301 C St, Ste 1400, Sacramento, CA 95816 USA; 2 Department of Dermatology, University of New Mexico School of Medicine, Albuquerque, NM USA; 3 Department of Dermatology, Keck School of Medicine at University of Southern California, Los Angeles, CA USA; 4 Department of Dermatology, University of California, Davis, School of Medicine, Sacramento, CA USA; 5 Department of Biological Sciences, California State University, Sacramento, CA USA; 6 College of Medicine, California Northstate University, Elk Grove, CA USA; 7 Pacific Skin Institute, Sacramento, CA USA

**Keywords:** Cutaneous closure technique, Cutaneous surgery, Scar evaluation, Undermining, Wound tension

## Abstract

Undermining is thought to improve wound outcomes; however, randomized controlled data regarding its efficacy are lacking in humans. The objective of this randomized clinical trial was to determine whether undermining low to moderate tension wounds improves scar cosmesis compared to wound closure without undermining. Fifty-four patients, 18 years or older, undergoing primary linear closure of a cutaneous defect with predicted postoperative closure length of ≥ 3 cm on any anatomic site were screened. Four patients were excluded, 50 patients were enrolled, and 48 patients were seen in follow-up. Wounds were divided in half and one side was randomized to receive either no undermining or 2 cm of undermining. The other side received the unselected intervention. Three months, patients and 2 masked observers evaluated each scar using the Patient and Observer Scar Assessment Scale (POSAS). A total of 50 patients [mean (SD) age, 67.6 (11.5) years; 31 (64.6%) male; 48 (100%) white] were enrolled in the study. The mean (SD) sum of the POSAS observer component scores was 12.0 (6.05) for the undermined side and 11.1 (4.68) for the non-undermined side (*P* = .60). No statistically significant difference was found in the mean (SD) sum of the patient component for the POSAS score between the undermined side [15.9 (9.07)] and the non-undermined side [13.33 (6.20)] at 3 months. For wounds under low to moderate perceived tension, no statistically significant differences in scar outcome or total complications were noted between undermined wound halves and non-undermined halves.

**Trail Registry**: Clinical trials.gov Identifier NCT02289859. https://clinicaltrials.gov/ct2/show/NCT02289859.

## Introduction

Surgical undermining is a popular technique used in cutaneous surgery, often to decrease tension during wound closure. Various undermining techniques exist, from electrosurgical methods to blunt and sharp undermining using cold steel instruments, the latter of which is used more frequently in dermatologic surgery [1].

Peripheral undermining of the sides and tips of fusiform wounds can push the skin away in a horizontal plane and minimize vertical tissue protrusions at the ends [2]. It has also been theorized that the plate-like scar created as a result of undermining all quadrants of a wound allows for even scar contracture, minimizing scar spread and standing cone formation [1]. Undermining is an effective way to separate the skin bordering a wound bed from the fibrous bands adjacent to underlying subcutaneous tissue that may limit its movement [1, 3]. Adequate tissue laxity is vital for wound closure in order to reduce the risk of ischemic necrosis, spreading scars and suture trauma [4]. Despite its many purported benefits, undermining can also cause decreased dermal perfusion and resulting necrosis or poor flap viability, hematomas, traumatic alopecia, sensory and motor nerve damage and poor wound cosmesis [1, 5–7].

Though undermining is widely used, randomized controlled data determining whether this surgical maneuver improves outcomes are limited. Studies in animal models have demonstrated benefits of undermining on reducing wound tension, but the focus has been more on wound biomechanics, rather than scar appearance [3, 5, 8, 9]. In one human study, undermining was shown to improve cosmetic result of circular wounds that were allowed to heal by secondary intention. However, to our knowledge, there are no randomized controlled studies assessing the effects of undermining on primary linear closure of cutaneous defects [10].

Given that the use of undermining for high-tension wounds is seen as a necessity by most authorities, we sought to investigate its utility in wounds of low to moderate tension, a subset of patients where more clinical equipoise exists. In order to control for confounders including, age, gender, race, and location, a split wound trial was undertaken.

## Methods

### Study design

This prospective, 2-arm, randomized, evaluator-blinded registered (Clinicaltrials.gov identifier: NCT02289859) clinical trial took place at the University of California, Davis outpatient academic dermatology clinic. Patients were continuously enrolled between October 2014 and November 2014 with follow-up completion in February 2015. Ethical approval was obtained through the University of California, Davis Institutional Review Board before study commencement, and all patients provided verbal and written consent to enrollment. To minimize the number of uncontrolled variables, a split scar model was used, in congruence with past studies that have looked at cuticular suturing techniques [11–13].

### Patient eligibility and a priori power analysis

Patients were eligible if they were 18 years or older, able to give informed consent, and undergoing primary linear closure of cutaneous defect with predicted postoperative closure length of greater than 3 cm on any anatomic site. Wounds more than 3 cm in diameter or on the scalp were excluded as we sought only to study outcomes in low to moderate tension closures. An a priori power analysis with 90% power and an alpha of 0.05 indicated that 50 patients would need to be enrolled to detect a 3-point difference in the POSAS scale, assuming a standard deviation (SD) of 6 (pmid 25,619,206) and an attrition rate of 20%.

### Randomization, allocation, concealment, and interventions

The surgical wounds were divided in half and labeled as “A” and “B,” with “A” always superior relative to the patient or on the left side from the surgeon’s perspective and “B” the opposite of “A.” A randomization list was generated before study recruitment from the website random.org. This list was then transferred in an excel file and uploaded onto the randomization module of a web-based study data capture system (REDCap) [14] by a clinic staff member uninvolved in recruitment, intervention, and assessment. One side was randomized to receive no undermining and the other side to receive 2 cm of undermining. Undermining was performed using the sharp technique which allows better control of the plane of dissection than blunt undermining.^1^All undermining was performed in the superficial subcutaneous plane, to minimize chances of motor nerve damage. [1] The size of the suture material and placement interval for wound closure was determined by the individual surgeon but was kept the same for both sides of the wound. Wound closure was accomplished in standard bilayered fashion with buried vertical mattress sutures using polyglactin 910 for deep sutures and 5–0 fast absorbing gut for cuticular sutures. After suturing was completed, white petrolatum ointment was applied to the entire length of the wound, followed by a sterile pressure dressing. Wound care instructions were given in verbal and written format. Patients were instructed to avoid strenuous activity for one week, to change their dressings daily, and to apply petrolatum ointment using a cotton-tipped applicator to the entire wound daily for 1 week or until the wound was fully healed. Follow-up arrangements were made for 3 months for study purposes and routine patient care.

### Assessments

The primary outcome of cosmetic appearance of the scar, as determined by the sum of the components of the observer portion of the Patient and Observer Scar Assessment Scale (POSAS), was evaluated 3 months after surgery. Assessments of surgical scars at 3 months are at least moderately correlated with those at 12 months, and differences in interventions tend to diminish with time. [15–17] Thus, if no difference in outcomes is seen at 3 months, it is unlikely any will be seen at a later timepoint. Secondary outcomes included the sum of the components of the patient POSAS assessments, scar width (measured at 1 cm from the midpoint of the scar for both halves), and complication rates.

The POSAS [15] is a validated outcome instrument for assessing outcomes of scars. The POSAS is based on a 10-point scoring system with 1 representing normal-appearing skin and 10 representing the worst scar imaginable. The total score ranges from 6 to 60 with a lower score representing more normal appearing skin on the scale. The POSAS has been validated when 2 independent observers are used [18] and has been used in numerous surgical studies [12, 13, 17, 19–25]. The scar outcome was evaluated in person by the patient and 2 blinded observers who were not present during the intervention.

The REDCap^4^ electronic web-based data capture tool, hosted by The University of California Davis Medical Center, was used to capture and manage study data.

### Statistical analysis

Data were examined based on an intention-to-treat analysis. Summary statistics were used to describe the baseline demographic and clinical characteristics of the patient population. Pairwise comparisons were used at 3 months after the procedure to analyze the differences between the use of undermining and no undermining of wounds in investigator scar assessment, patient scar assessment, and surgical complications.

The Wilcoxon matched-pairs signed-rank test was used to determine the equality of matched pairs of observations for surgical outcome variables, which were binary. The null hypothesis of this test is that both distributions are the same. For the continuous outcomes of investigator-assessed and patient-assessed scar appearance and symptoms, a paired *t* test was used to compare the differences between portions of the wound which were undermined versus those that were not undermined. All results achieving *P* < 0.05 (two-tailed) were considered statistically significant. The analyses were performed with STATA/MP 13 (College Station, Texas).

## Results

**Fig. 1:**
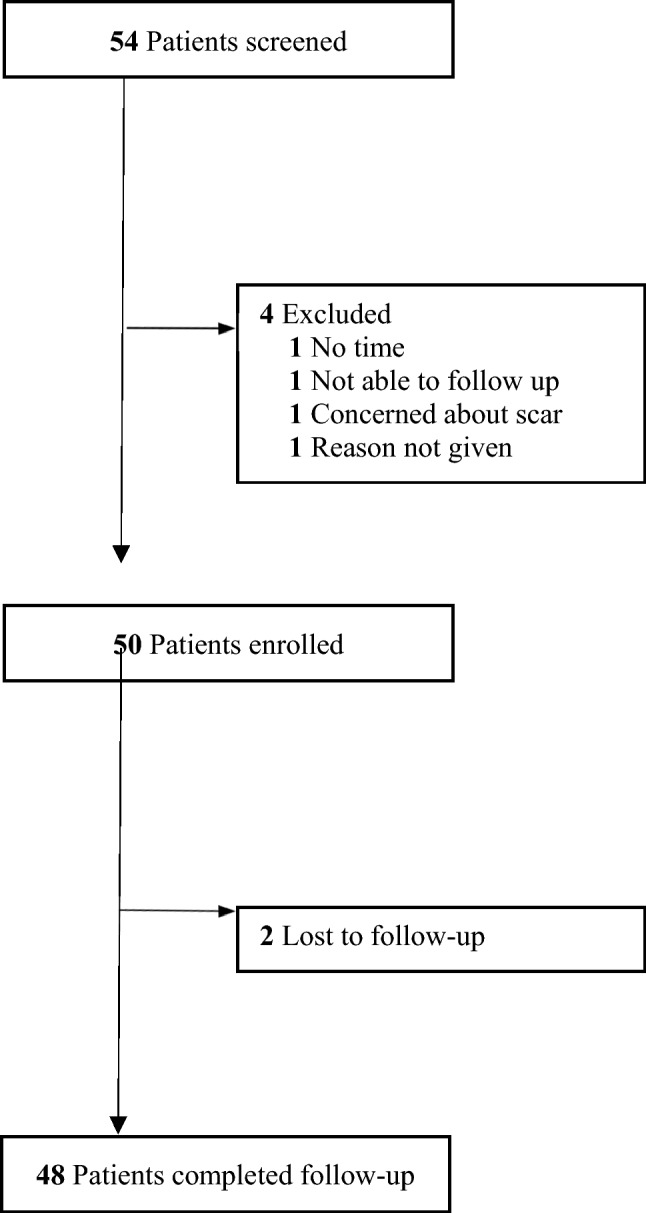
CONSORT Diagram. Screening, enrollment, and follow-up of study patients

Fifty-four patients were screened and 50 were enrolled to achieve the recruitment goal of 50 patients (Table 1). Forty-eight patients (96%) returned for follow-up (Fig. 1). Thirty-one subjects were male (64.6%) and 100% were white. A fellowship trained dermatologic surgeon performed the study intervention in 14 (29.2%) cases, a dermatologic surgery fellow in 15 (31.3%) cases, and a dermatology resident under direct supervision of a fellowship trained dermatologic surgeon in 19 (39.6%) cases. Thirty-three sites were on the head or neck (66%), with a mean wound length of 5.4 cm (Table 1).

**Table 1:** Baseline characteristics of study population and surgical procedure data

Characteristics	Total
Patients/surgical sites, No	
Enrolled	50
Completed	48
Mean age, y (SD)	67.6(11.5)
Sex, No. (%)	
Male	31(64.6)
Female	17(35.4)
Race, No. (%)	
White	48(100)
Procedure type, No. (%)	
Mohs	32(75.0)
Excision	16(33.3)
Surgical site, No. (%)	
Forehead	5(10.4)
Temple	6(12.5)
Preauricular	4(8.3)
Cheek	6(12.5)
Chin	1(2.1)
Neck	11(22.9)
Chest	1(2.1)
Back	6(12.5)
Shoulder	3(6.3)
Arm	4(8.3)
Leg	1(2.1)
Wound closure length, mean (SD), cm	5.4(1.5)
Mean follow-up time, mean (SD), months	3.2(0.36)
Surgeon, No. (%)	
Attending	14(29.2)
Fellow	15(31.3)
Resident	19(39.6)

For the primary outcome measure, the mean sum of the POSAS component scores of the blinded reviewers, there were no significant differences between the undermined area [12.0 (SD 6.05)] and the non-undermined area [11.1 (SD 4.68)] (Table 2). There were no significant differences between the two interventions at 3 months for all of the subcategory scar outcomes such as vascularity, pigmentation, thickness, relief, pliability, surface area, and overall blinded observer opinion as well as the mean patient POSAS scores for pain, itching, color, stiffness, thickness, irregularity, and overall opinion. (Table 2). There was also no statistically significant difference between the mean width of the undermined side [1.29 mm (SD 1.11)] and the mean width of non-undermined side [1.22 mm (SD 1.17)] (*P* = 0.65).

**Table 2:** Observer and patient POSAS scores and scar width at 3-month follow-up

Scar assessment	POSAS score mean (SD)		*P* value
	Undermining side	No undermining side	
Observer POSAS, mean (SD)			
Vascularity	2.28(1.60)	2.18(1.27)	0.6634
Pigmentation	1.39(0.78)	1.35(0.60)	0.8924
Thickness	1.93(1.27)	1.66(0.89)	0.1016
Relief	2.19(1.37)	2.05(1.13)	0.5814
Pliability	1.85(1.25)	1.66(0.92)	0.3020
Surface area	2.38(1.46)	2.25(1.18)	0.7039
Total POSAS	12.0(6.05)	11.1(4.68)	0.4205
Overall opinion	2.31(1.19)	2.34(1.19)	0.6005
Patient POSAS, mean (SD)			
Pain	1.32(1.14)	1.04(0.29)	0.0964
Itching	1.11(0.37)	1.19(0.61)	0.2093
Color	3.72(2.56)	3.40(2.25)	0.6521
Stiffness	3.72(2.80)	2.83(1.97)	0.0876
Thickness	2.68(1.98)	2.13(1.51)	0.0893
Irregularity	3.51(2.76)	2.74(2.14)	0.0854
Total POSAS	15.88(9.07)	13.33(6.20)	0.0832
Overall impression	3.15(2.45)	2.77(2.00)	0.4663
Scar width^*^, mean (SD), mm	1.29(1.11)	1.22(1.17)	0.6541

*SD* standard deviation, *POSAS* patient observer scar assessment scale

^*^The scar width was measured 1 cm from midline

There were no significant differences in complication rates for either intervention at 3-month follow-up (Table 3). There were 3 cases of infection on the undermined side and 2 cases of infection on the non-undermined side. Two of these sides affected by infection were from one participant. Another participant experienced a hematoma of the entire wound. There was also 1 case of bleeding of the undermined side and 2 cases of suture granuloma noted of the non-undermined side. Wound dehiscence, seroma, uneven edges, and wound contour abnormalities were not observed.

**Table 3:** Complications at 3-month follow-up

Complication	Incidence (%)		*P* value
	Undermined side	Non-undermined side	
Infection	3	2	0.3173
Hematoma	1	1	–^*^
Other	1^a^	2^b^	0.5637

^a^Bleeding

^b^Spitting suture

^*^P value could not be calculated when the complication occurred in the same patient

## Discussion

For wounds under perceived low to moderate tension, there was no statistically significant difference in the appearance of scars resulting from primary closure of cutaneous defects when using undermining versus not undermining the wound as rated by 2 blinded observers and the participants of the study. Complications also did not significantly differ between the two intervention arms.

Undermining is widely considered an important technique in cutaneous surgery with many espousing its benefits. However, most data supporting its benefits come from animal studies. A study in a guinea pig model showed that undermining prevented the development of trapdoor deformities in undermined circular wounds repaired with transposition flaps versus those which were not undermined [26]. Studies done in pig models have found that wound undermining reduces skin tension when compared to imbrication and intraoperative expansion [5, 8].

The extent of wound undermining may also have varying effects. Cox et al. found a difference in the effects of undermining on narrowly and broadly based flaps in pig models [3]. They demonstrated that as the area of undermining is increased, the force of advancing the edge of a flap in narrowly based flaps decreases, whereas there is an increase in shearing force for broadly based flaps that is proportionate to the circumference of the area undermined [3].

Quantitative effects of undermining have also been demonstrated in human patients undergoing scalp reduction surgery for the correction of male pattern baldness [27]. Undermining of scalp flaps in the subgaleal plane at ranges of 1, 5, and 15 cm laterally in both directions from a longitudinal incision of a reversed Y scalp incision showed that the greatest decrease in the tension required for flap advancement occurred with undermining in the 5 cm range. Additional undermining did not significantly decrease the tension required to advance the flap [27].

We have only identified a single study, other than ours, that addresses the cosmetic effects of undermining in human subjects [10]. In this randomized clinical trial by Huang et al. circular wounds less than 2 cm in diameter resulting from excision of benign lesions were randomized into a non-undermined group versus wounds which were undermined at a range of 5, 10, and 15 mm. All wounds were allowed to heal by secondary intention. There were no statistically significant differences in the rates of complications between groups. A statistically significant difference was found in scar width between the control and 10 mm range undermining group. Scar appearance as rated by the Visual Assessment of Linear Scars (VALS) tool showed an improvement as the range of undermining increased, but no statistically significant difference was found between the 10 and 15 mm undermining group.

Our results demonstrate findings different to what is commonly advocated in the literature. This may be related to the smaller excisions performed in our study and the resulting decreased tension that would not necessarily require undermining. Not undermining during wound closure may help decrease operative time as well as the possible adverse effects of undermining. While undermining may be of more importance in larger wounds and/or wounds that are under greater tension, our study does not address this situation.

There were many strengths of this study including a priori power analysis, true randomization, blinded observer assessment, allocation concealment, use of a validated outcome instrument, low attrition rate as well as surgeries performed by physicians with a variety of experience levels. Surgical sites were also done on various parts of the body.

Limitations of this study include that it was done at a single center with participants who were primarily elderly Caucasian males. Additionally, the optimal amount of undermining is not known. We used 2 cm, but more or less undermining may have resulted in different outcomes. Clearly, our study does not address wounds under high tension, where undermining would theoretically have the most potential benefits. Finally, as in clinical practice, no scientific method was used to measure wound tension.

## Conclusion

There was no significant difference in scar outcome between undermined side and non-undermined side of wounds under moderate to low perceived tension. Complication rates were similar for both interventions.

## Data Availability

The data from this study are available upon request from the corresponding author.

## References

[1] Chen DL, Carlson EO, Fathi R, Brown MR (2015). Undermining and hemostasis. Dermatol Surg.

[2] Zitelli JA (1990). TIPS for a better ellipse. J Am Acad Dermatol.

[3] Cox KW, Larrabee W (1982). A study of skin flap advancement as a function of undermining. Arch Otolaryngol.

[4] Cohen BH (1995). Noninvasive undermining. Dermatol Surg.

[5] Krishnan NM (2016). Reducing wound tension with undermining or imbrication-do they work?. Plast Reconstr Surg Glob Open.

[6] Melis P, Noorlander ML, van der Horst CM, van Noorden CJ (2002). Rapid alignment of collagen fibers in the dermis of undermined and not undermined skin stretched with a skin-stretching device. Plast Reconstr Surg.

[7] Boyer JD, Zitelli JA, Brodland DG (2001) Undermining in cutaneous surgery. Dermatol Surg 27(1): 75–78. Available: https://www.ncbi.nlm.nih.gov/pubmed/11231251 11231251

[8] Mackay DR, Saggers NK, Manders EK (1990) Stretching skin: undermining is more important than intraoperative expansion. Plast Reconstr Surg 86(4): 722–730. Available: https://www.ncbi.nlm.nih.gov/pubmed/2217588 2217588

[9] McGuire MF (1980) Studies of the excisional wound: I. Biomechanical effects of undermining and wound orientation on closing tension and work. Plast Reconstr Surg 66(3): 419–427. Available: https://www.ncbi.nlm.nih.gov/pubmed/7422728 7422728

[10] Huang L, Wenzhi L (2014) The role of the undermining during circular excision of secondary intention healing. Am Surg 80(6): 587–594. Available: https://www.ncbi.nlm.nih.gov/pubmed/24887797. 24887797

[11] Moody BR, McCarthy JE, Linder J, Hruza GJ (2005) Enhanced cosmetic outcome with running horizontal mattress sutures. Dermatol Surg 31(10): 1313–1316. Available: https://www.ncbi.nlm.nih.gov/pubmed/16188185. 10.1111/j.1524-4725.2005.31209 16188185

[12] Wang AS (2014). Set-back versus buried vertical mattress suturing: results of a randomized blinded trial. J Am Acad Dermatol.

[13] Custis T, Armstrong AW, King TH, Sharon VR, Eisen DB (2015). Effect of adhesive strips and dermal sutures vs dermal sutures only on wound closure: a randomized clinical trial. JAMA Dermatol.

[14] Harris PA, Taylor R, Thielke R, Payne J, Gonzalez N, Conde JG (2009). Research electronic data capture (REDCap)–a metadata-driven methodology and workflow process for providing translational research informatics support. J Biomed Inform.

[15] Draaijers LJ et al (2004) The patient and observer scar assessment scale: a reliable and feasible tool for scar evaluation. Plast Reconstr Surg 113(7): 1960–1965. Available: https://www.ncbi.nlm.nih.gov/pubmed/15253184 10.1097/01.prs.0000122207.28773.56 15253184

[16] Alam M, Posten W, Martini MC, Wrone DA, Rademaker AW (2006). Aesthetic and functional efficacy of subcuticular running epidermal closures of the trunk and extremity: a rater-blinded randomized control trial. Arch Dermatol.

[17] Kappel S (2015). Does wound eversion improve cosmetic outcome?: results of a randomized, split-scar, comparative trial. J Am Acad Dermatol.

[18] Eom JM, Ko JH, Choi JS, Hong JH, Lee JH (2013). A comparative cross-sectional study on cosmetic outcomes after single port or conventional laparoscopic surgery. Eur J Obstet Gynecol Reprod Biol.

[19] Jina H, Simcock J (2011) Median sternotomy scar assessment. N Z Med J 124(1346): 57–62. Available: https://www.ncbi.nlm.nih.gov/pubmed/22143853. 22143853

[20] Mosterd K, Arits AH, Nelemans PJ, Kelleners-Smeets NW (2013). Aesthetic evaluation after non-invasive treatment for superficial basal cell carcinoma. J Eur Acad Dermatol Venereol.

[21] Zhuang AR, Beroukhim K, Armstrong AW, Sivamani RK, Eisen DB (2019). Comparison of 2-octylcyanoacrylate versus 5–0 fast-absorbing gut during linear wound closures and the effect on wound cosmesis. Dermatol Surg.

[22] Sklar LR, Pourang A, Armstrong AW, Dhaliwal SK, Sivamani RK, Eisen DB (2019). Comparison of running cutaneous suture spacing during linear wound closures and the effect on wound cosmesis of the face and neck: a randomized clinical trial. JAMA Dermatol.

[23] Pourang A, Crispin MK, Clark AK, Armstrong AW, Sivamani RK, Eisen DB (2019). "Use of 5–0 fast absorbing gut vs 6–0 fast absorbing gut during cutaneous wound closure on the head and neck: a randomized evaluator-blinded split-wound comparative effectiveness trial. J Am Acad Dermatol.

[24] Custis T, Armstrong AW, King TH, Sharon VR, Eisen DB (2015). Effect of adhesive strips and dermal sutures vs dermal sutures only on wound closure: a randomized clinical trial. JAMA Dermatol.

[25] Joo J (2014). Purse-string suture vs second intention healing: results of a randomized blind clinical trial. JAMA Dermatol.

[26] Kaufman AJ, Kiene KL, Moy RL (1993) Role of tissue undermining in the trapdoor effect of transposition flaps. J Dermatol Surg Oncol 19(2): 128–132. Available: https://www.ncbi.nlm.nih.gov/pubmed/8429138. 10.1111/j.1524-4725.1993.tb03441.x 8429138

[27] Raposio E, Nordstrom RE, Santi PL (1998). Undermining of the scalp: quantitative effects. Plast Reconstr Surg.

